# 113. Upstream with the Paddle: Wastewater-Based Genomic Surveillance and Near-Source Detection of Vancomycin-Resistant Enterococcus Prior to Case Identification

**DOI:** 10.1093/ofid/ofaf695.045

**Published:** 2026-01-11

**Authors:** Valerie J Morley, Casandra Philipson, Paige Salerno, Nora Watson, Marleen M Welsh, Michael Backlund, Tyler Moeller, Amanda Smith, Dawn Gratalo, Beth Higa Roberts, Andrew Sum, Alexandra M Simas, Tisza A S Bell, Diego Insausti, Benjamin Knisely, Melissa Austin, Paige E Waterman, Wesley Campbell

**Affiliations:** Ginkgo Biosecurity, Albuquerque, NewMexico; Ginkgo Bioworks, Boston, Massachusetts; Ginkgo Bioworks, Boston, Massachusetts; Walter Reed National Military Medical Center, Bethesda, Maryland; Booz Allen Hamilton, Bethesda, Maryland; WRNMMC, Bethesda, Maryland; Naval Medical Research Unit 6, Maryland, Maryland; Walter Reed National Military Medical Center, Bethesda, Maryland; Ginkgo Bioworks, Boston, Massachusetts; Ginkgo Biosecurity, Albuquerque, NewMexico; Ginkgo Bioworks, Boston, Massachusetts; Ginkgo Bioworks, Boston, Massachusetts; Booz Allen Hamilton, Bethesda, Maryland; BOOZ ALLEN HAMILTON, MIAMI SPRINGS, Florida; Booz Allen Hamilton, Bethesda, Maryland; Walter Reed National Military Medical Center, Bethesda, Maryland; USUHS, Bethesda, Maryland; Walter Reed National Military Medical Center, Bethesda, Maryland

## Abstract

**Background:**

Wastewater surveillance (WWS) is a public health tool complementing traditional case reporting when deployed to near-source sites. Presented here is a cluster event involving oncology patients experiencing vancomycin-resistant enterococci (VRE) infection where active WWS protocols served as a leading detection tool for an antimicrobial resistant (AMR) pathogen of significant clinical impact to immunocompromised patients.

**Figure:**
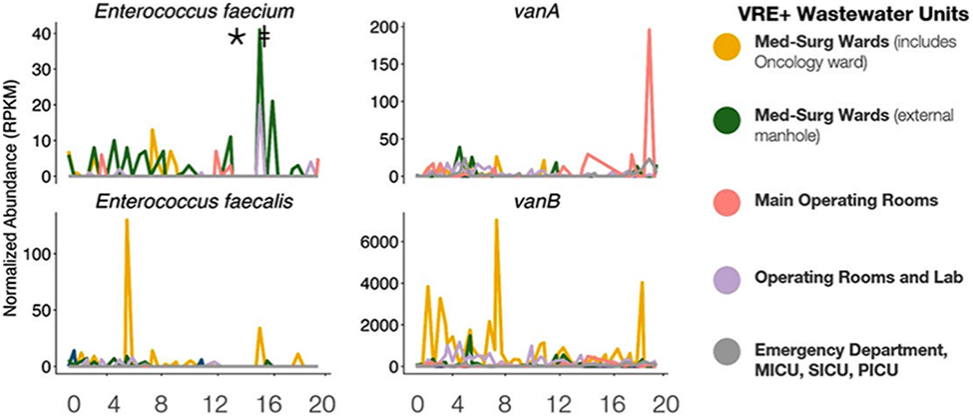
Plots Depicting Longitudinal Normalized Abundance of VRE and AMR genes (vanA, vanB) in Wastewater from Hospital Buildings. * VRE positive cultures patient 1; ^†^ VRE positive cultures patient 2; RPKM: Reads Per Kilobase Per Million Mapped Reads; VRE: Vancomycin Resistant Enterococcus; AMR: Antimicrobial Resistance; Med-Surg: Medical-Surgical Inpatient Wards; MICU: Medical Intensive Care Unit; SICU: Surgical Intensive Care Unit; PICU: Pediatric Intensive Care Unit

**Figure:**
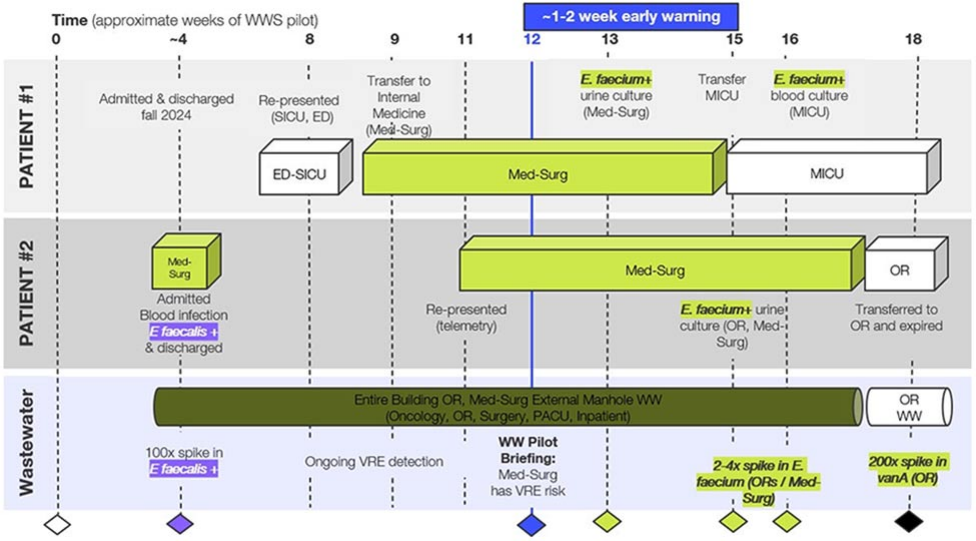
Building Level Wastewater and Clinical Event Timelines WWS: Wastewater Surveillance; ED: Emergency Department; SICU: Surgical Intensive Care Unit; Med-Surg: Medical Surgical Ward/Building; OR: Operating Room (location or building); PACU: Post Anesthesia Care Unit

**Methods:**

WWS sampling was conducted 3-times weekly from October 2024 through April 2025 at 8 outpatient, inpatient, and MEDEVAC housing effluent points. Samples were concentrated, nucleic acid was extracted, and over 180 bacterial pathogens and 1200 AMR gene markers were analyzed using specialized bioinformatic pipelines. Clinical isolates underwent whole genome sequencing (WGS) as part of the Department of Defense Multidrug-resistant organism Repository and Surveillance Network (MRSN).

**Results:**

Building-level WWS inclusive of the oncology ward identified a spike in VRE one week before two clinical cases were reported in inpatient oncology patients (Figure 1). An alert (time 0) was registered to the facility. A positive urine culture from patient 1 corresponded with a 4-fold increase in *Enterococcus faecium* and vanA gene detection in wastewater (week 1). Patient 2 tested positive by urine culture for the same pathogen (week 2) (Figure 2). MRSN WGS analysis of the patients’ clinical isolates obtained during week 3 and 4 confirmed the case cluster, while genomic fragments of the clinically relevant organisms detected by WWS during the outbreak indicated a detection in the weeks prior. The Infection Prevention and Control Team (IPaC) investigation suspected shared care teams contributed to the cluster event. Patient 2, admitted from a nursing facility earlier in the month, is the suspected reservoir.

**Conclusion:**

Demonstrating the operational value of our unique, building-level, near-source WWS protocol to detect pathogen hot spots before clinical recognition, the study team provided early awareness as designed. Furthermore, this study exemplifies how WWS can complement genomic analysis of clinical isolates as part of a layered IPaC and antimicrobial stewardship effort to improve antimicrobial prescribing practices, patient safety, and outcomes.

**Disclosures:**

All Authors: No reported disclosures

